# Phenyl Acid Induced Inhibition of Methanogenesis in CO_2_ Reducing Organisms

**DOI:** 10.1111/1758-2229.70082

**Published:** 2025-03-12

**Authors:** Mathias Wunderer, Martin Unterkircher, Anna Schmidhofer, Eva Maria Prem, Andreas O. Wagner

**Affiliations:** ^1^ Universität Innsbruck Department of Microbiology Innsbruck Austria

**Keywords:** anaerobic digestion, aromatic compound, biogas, hydrogenotrophic methanogen, methanogenic archaea

## Abstract

During anaerobic digestion (AD) of lignocellulose‐ and protein‐rich substrates known to contain a high load of aromatic compounds, various undesired intermediates can arise, which can accumulate and cause serious disturbances during the cascade‐like AD process. The phenyl acids phenyl‐acetic‐(PAA), phenyl‐propionic‐(PPA), and phenyl‐butyric acid (PBA) are such intermediates suspected to negatively affect the microbial community, resulting in a decreased biogas yield. In the present study, the impact of PAA, PPA, and PBA on the metabolism of CO_2_ reducing methanogens was investigated. The mesophilically incubated *Methanococcus vannielii* and *Methanospirillum hungatei* showed a higher sensitivity than the thermophilic *Methanoculleus thermophilus* , *Methanothermobacter thermoautotrophicus*, and *Methanothermobacter wolfei*. A concentration of 35 mM PPA and 35 mM PBA inhibited methanogenesis and the growth of *M. hungatei* almost completely. PBA had the greatest detrimental impact on methanogens across all tested phenyl acids, followed by PPA and PAA. However, in further investigations, it has to be clarified if and how other microorganisms involved in AD are impacted by phenyl acids. A more detailed knowledge will help to better understand disturbances due to phenyl acid emergence caused by the degradation of lignocellulose‐ and protein‐rich substrates, to ensure a stable AD process even at high loads of these substrates.

## Graphical abstract

In the presented study, the impact of phenyl acids on hydrogenotrophic methanogenic pure cultures was evaluated. Mesophilic‐incubated methanogens showed a higher sensitivity against phenyl acids than thermophilic‐incubated methanogens. Phenyl‐butyric acid had the highest adverse effect on the metabolism of the tested methanogens, and 35 mM phenyl‐propionic‐ and phenyl‐butyric acids could prevent the metabolic activity of certain methanogenic pure cultures.

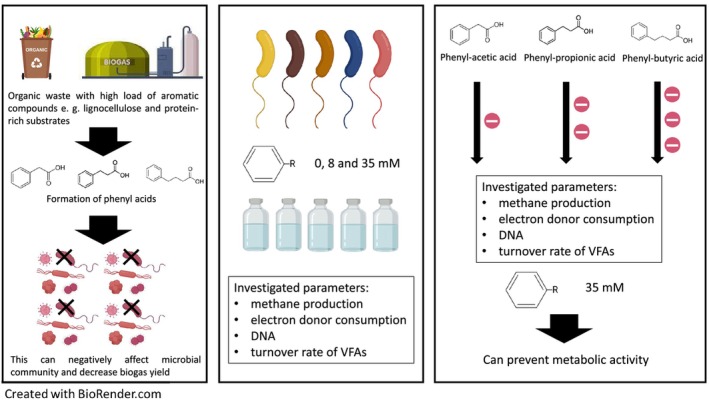

## 1 Introduction

The global energy demand is rising steadily; from 2012 to 2022, it has increased from 527 to 604 exajoules of primary energy consumption, corresponding to an annual growth of 1.4% (Energy Intitute 2023). Fossil energy carriers still hold the largest share of global primary energy carriers. In 2022, 32% of the globally used primary energy derived from oil, followed by coal at 27% and natural gas at 23%. Compared to these, non‐fossil energy carriers are far less in use, and only 4% of worldwide energy demand is derived from nuclear energy, 7% from hydroelectricity, and 8% from other renewables (Energy Institute 2023). To curb climate change, the European Union wants to reduce greenhouse gas emissions by 55% by 2030 and by 100% by 2050 (European Parliament n.d.). To achieve these targets, we must significantly reduce the use of fossil energy carriers, extend the spectra of renewable and climate‐neutral energy carriers, and increase their capacity. Renewable energy carriers include solar power, wind power, hydroelectricity, geothermal heat, and biogas from biomass.

To produce biogas, various organic matter can be anaerobically digested. This can directly be used to generate heat or electricity or be upgraded through different methods to biomethane (> 96% methane) and fed into the gas grid, which represents an ideal storage system. In the European Union and the United Kingdom, energy crops still hold a large share of feedstock used for biogas production at 42%, followed by agricultural residues at 24%, biowaste at 16%, sewage sludge, and industrial waste at 8%, and 2% are attributable to other feedstocks (Fehrenbach et al. 2023). In terms of effective, sustainable, and ethical energy management, the use of energy crops has to be reduced significantly and that of agricultural residues and other biowaste like tree pruning or food waste has to be expanded.

The degradation of different organic wastes like agricultural, industrial, kitchen, and other food residues has a high potential for biogas production. During the degradation of these lignocellulose‐ and protein‐rich substrates, especially under overload conditions, different aromatic compounds like phenyl‐acetic acid (PAA), phenyl‐propionic acid (PPA), and phenyl‐butyric acid (PBA) can accumulate (Prem et al. 2019, 2021; Wagner et al. 2019; Hecht and Griehl 2009). This can negatively affect anaerobic digestion (AD) performance, resulting in reduced biogas production, volatile fatty acid (VFA) accumulation, and a potential shift in the methanogenic community from dominant acetoclastic to hydrogenotrophic methanogenesis (Prem et al. 2021; Cabrol et al. 2015; Prem et al. 2023; Prem et al. 2023). Prem et al. (2023) could also show that the used substrate is decisive for the impact of the phenyl acids on the AD community. When VFAs are present as sole carbon sources, the methane yield, microbial community diversity, and the extracellular polymeric substance (EPS) quantity and quality are lower than if microcrystalline cellulose is used as a substrate (Prem et al. 2023). This indicates that various trophic groups in AD systems are impacted differently and that EPS expression seems to protect against the negative effects of phenyl acids. According to Sierra‐Alvarez and Lettinga (1991), the 50% and 80% inhibitory concentrations of PAA for acetoclastic methanogenesis are already reached at 5.27 and 9.18 mM, respectively. However, in different other studies, a direct negative effect on the methanogenic community of AD systems could not be proven (Prem et al. 2019, 2020; Wagner et al. 2019; Hecht and Griehl 2009). These are contradictory results and need to be tested to gain a better understanding of the impact of phenyl acids on the methanogenic community, to enable a stable AD process from substrates with a high lignocellulose or protein content, the main precursors of phenyl acids (Prem et al. 2019; Wagner et al. 2019; Hecht and Griehl 2009; Glissmann et al. 2005). To elucidate the influence of the phenyl acids on methanogenic Archaea, it is of utmost importance to take a step back and test the effect of phenyl acids on methanogens in pure culture, excluding complex microbial interactions.

In the present study, the effect of the phenyl acids phenyl‐acetic acid, 3‐phenyl‐propionic acid, and 2‐phenyl‐butyric acid on methanogenesis, and therefore, the energy conservation and growth of different methanogenic pure cultures was tested.

## 2 Materials and Methods

### 2.1 Experimental Setup and Sampling

Five methanogenic Archaea were cultivated in five replicates each in DSMZ 119 medium or DSMZ 141 medium, respectively, using serum flasks (please refer to Section 2.2). Media preparation under strictly anaerobic conditions followed the instructions given by Wagner, Markt et al. (2019). Different concentrations (0, 10, and 35 mM) of the phenyl acids PAA, 3‐PPA, and 2‐PBA were added to the media to explore the effect of these phenyl acids on growth and methanogenetic activity. Samples were taken at five or six different time points, respectively, following the growth dynamics of the methanogens as determined in preceding experiments using methane production kinetics. To evaluate methane production, the overpressure in the serum flask was measured with a manometer before gas samples (1.14 mL each) were taken and immediately analysed via gas chromatography (H_2_, CO_2_, and CH_4_). Liquid samples were taken (1 mL), immediately frozen, and stored at −18°C before they were used for further analyses. The 1 mL liquid samples were centrifuged at 11,000 × *g* for 10 min, and 600 μL of the supernatant was used for VFA analyses and the pellet for DNA extraction.

### 2.2 Cultivation

Cultures of *Methanococcus vannielii* SB (DSM 1224), *Methanospirillum hungatei* JF‐1 (DSM 864), *Methanoculleus thermophilus* (DSM 2373), *Methanothermobacter thermoautotrophicus* Delta H (DSM 1053), and *Methanothermobacter wolfei* (2970) were obtained from DSMZ‐Deutsche Sammlung von Mikroorganismen und Zellkulturen (Braunschweig, Germany). All tested methanogens were cultivated in 250 mL serum flasks and filled with 50 mL of respective DSMZ medium (*M. vannielii, M. hungatei*, *M. thermoautotrophicus, and M. wolfei* in DSMZ 119 medium; *M. thermophilus* in DSMZ 141 medium). Depending on the tested methanogen, a volume of 195 or 260 mL H_2_‐CO_2_ (80:20 vol/vol) was added after inoculation, whereby the different parallels and variants of the respective methanogen did not differ by more than ±25 mbar in pressure. *M. vannielii and M. hungatei* were incubated at 37°C, while *M*. *thermoautotrophicus, M. thermophilus*, and *M. wolfei* were incubated at 52°C. All cultures were inoculated with 10% (vol/vol) cell suspension.

### 2.3 Gas Analysis via GC

Overpressure in the headspace of the serum flask was measured with a manometer (GDH 200‐13, Greisinger electronic, Germany), taking the actual air pressure as a reference (www.zamg.ac.at). The composition of the biogas (H_2_, CH_4_, CO_2_) was determined with a GC 2010 gas chromatograph (Shimadzu, Japan) according to Wagner et al. (2011). CH_4_ production, as well as H_2_ consumption, was calculated according to Wunderer et al. (2022).

### 2.4 VFA Analysis via HPLC

The analysis of the VFAs formate and acetate was done with a Prominence HPLC system (Shimadzu, Japan). The separation of the VFAs occurred on a Rezex RFQ‐Fast Acid H+ (8%) 100 × 7.8 mm column (Phenomenex, Germany) and 5 mM H_2_SO_4_ as the mobile phase, whereas detection took place with a UV/VIS detector at 210 nm according to Wagner et al. (2017).

### 2.5 DNA Purification

DNA was extracted using the NucleoSpin Soil Kit (MACHERY&NAGEL, Germany) according to the manufacturer's recommendations and quantified with the Quant‐iT PicoGreen dsDNA Assay Kit (Invitrogen, USA) using an Anthos‐Zenyth Multimode Detector (Biochrom, Germany) as described previously (Wagner et al. 2015).

### 2.6 Calculation of Electron Flux

The calculation of electron turnover from the substrates formate (*M. vannielii* and *M. hungate* *i*) and H_2_‐CO_2_ into methane is based on the complete stoichiometric oxidation of these compounds into CO_2_ and water. 1 mol of consumed oxygen corresponds to four electron equivalents (e–q) (Markt et al. 2024). The final electron recovery in the produced methane was calculated on the incubation day when the respective control variant reached the maximum methane yield (please refer to Section 2.7) and is shown as a percentage of the initial substrate.

### 2.7 Statistical Analysis

**FIGURE 1:**
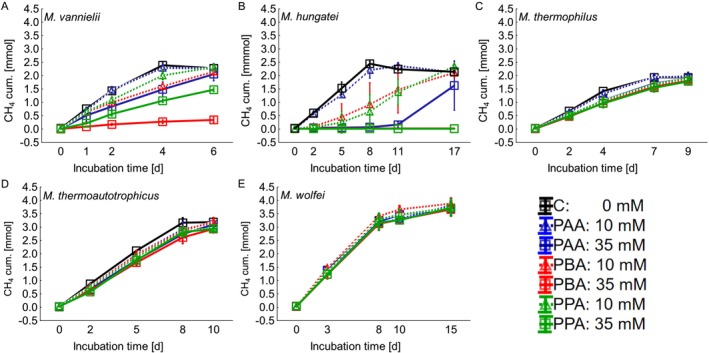
Cumulative methane production during the exposure to phenyl‐acetic acid (PAA), phenyl‐butyric acid (PBA), and phenyl‐propionic acid (PPA) in different concentrations (0, 10, and 35 mM): A = *Methanococcus vannielii* , B = *Methanospirillum hungatei* , C = *Methanoculleus thermophilus* , D = *Methanothermobacter thermoautotrophicus*, and E = *Methanothermobacter wolfei*.

Statistical analyses were carried out using STATISTICA 13 (TIBCO, USA). To evaluate the impact of the phenyl acids on methane production and growth of the tested methanogens, DNA and cumulative methane data from that incubation day, when the respective control variant reached the maximum methane yield, was used to test for normal distribution and homogeneity of variance and statistically compared to data of the same day in variants containing phenyl acids. Depending on the results of the Kolmogorov Smirnov and Lilliefors tests for normality (normal distribution) and the Levene test (homogeneity of variance) a one‐way ANOVA or Kruskal–Wallis ANOVA (non‐parametric) was carried out, respectively. According to Figure 1 maximum methane production was reached at day 4 in *M. vannielii* , day 8 in *M. hungatei* , day 7 in *M. thermophilus* , day 8 in *M. thermoautotrophicus*, and in *M. wolfei* , the control variant reached the maximum methane concentration on incubation day 15.

## 3 Results and Discussion

### 3.1 Methane Production

The cumulative methane production of the tested hydrogenotrophic methanogens is depicted in Figure 1. In the mesophilically incubated *M. vannielii* (Figure 1A), the control variant produced 2.39 mmol methane until day 4 and decreased again slightly until day 6. In the 10 mM PAA variant, the methane production was almost identical to the control and reached a 2.30 mmol methane until day 4. A concentration of 10 mM PPA led to a slight delay in methane production from the first day of the incubation period but produced the same amount of methane until day 6 (2.34 mmol CH_4_). This shows that 10 mM PPA did not decrease the methane yield but led to a delay in methane production. The same could be observed in the 10 mM PBA and 35 mM PAA variants. In samples containing 35 mM PBA and PPA, respectively, a strong delay in methane production was observed starting at day 0. While the variant with 35 mM PPA still produced 1.47 mmol methane until day 6, the variant with 35 mM PBA produced only 0.34 mmol methane. Compared with the control, methane production in the 35 mM PPA variant was delayed as reflected by a different methane production rate during the exponential phase (Figure 1A, please refer to the different slopes) and the maximum methane yield could be reached with an extended incubation period. In contrast, the low methane yield in the 35 mM PBA variant until day 6 shows that *M. vannielii* was strongly inhibited by this phenyl acid and that even with an extended incubation period, the methane yield from the control could not be reached. In *M. vannielii* , the control variant had a significantly higher methane yield than all other variants (Figure S1), except the 10 mM PAA variant. 10 mM PAA had a significantly higher methane yield than the 10 mM PBA variant and all 35 mM variants, whereas 10 mM PPA had a significantly lower methane content compared to the control variant and a significantly higher than all other variants, except the 10 mM PAA variant, for which no significant differences could be found. The addition of 35 mM PAA and 10 mM PBA, respectively, resulted in a significantly lower methane content than observed for controls, 10 mM PAA, and 10 mM PPA, and a significantly higher methane content than for 35 mM PBA and 35 mM PPA. The 35 mM PPA variant had a significantly higher methane yield than the 35 mM PBA variant and significantly lower than all other variants. Most inhibited was the 35 mM PBA variant, which had a significantly lower methane yield than all other tested variants.

Controls of the mesophilically incubated *M. hungatei* (Figure 1B) achieved a methane yield of 2.45 mmol after 8 days. Similar to results from *M. vannielii* , also with *M. hungatei* , the 10 mM PAA variant showed the highest similarity to the control and reached the maximum methane yield on day 8 with 2.22 mmol. In flasks containing 10 mM PBA and PPA, respectively, methane production started delayed on day 2. Nevertheless, both variants reached similar methane yields compared to the control when the incubation time was increased to 17 days. In contrast, the 35 mM PAA variant was completely inhibited at the beginning of the incubation period but started methane production at day 8, and still reached 1.63 mmol methane by day 17. The slope of the methane production indicates that with an extended incubation period, the methane yield of the control could have been reached. While in the 35 mM PBA and 35 mM PPA variants, methane production was almost completely inhibited, and only traces of methane could be produced throughout the whole incubation period. The control and 10 mM PAA variants had a significantly higher methane yield than the 35 mM PBA and PPA variants, while no significant differences could be found between all the other variants (Figure S1).

Also, in the thermophilically incubated *M. thermophilus* (Figure 1C), the 10 mM PAA variant showed almost identical methane production when compared to the control and reached 1.95 mmol methane until day 7 (1.94 mmol in the control). The kinetics in all the other variants were very similar but showed a slight delay in methane production starting with day 2. All other phenyl acid concentrations ended up with a mean of 1.62 ± 0.111 mmol methane until day 7. Even if there were only small differences in the methane content between the variants, significant differences were found (Figure S1). The control and 10 mM PAA variant had a significantly higher methane yield than all other variants.

Controls of the thermophilic *M. thermoautotrophicus* (Figure 1D) reached the maximum methane yield on day 8 at 3.16 mmol methane. The other variants showed a slight delay in methane production from day 2 on, but all of them could reach 2.79 ± 0.216 mmol methane by day 8. The Kruskal–Wallis ANOVA could not find any significant differences between the variants (Figure S1).

With the thermophilic *M. wolfei* (Figure 1E), no obvious effect of phenyl acids on methanogenesis could be observed. All variants exceeded 3.1 mmol methane until day 8, whereby the methane production rate decreased towards the end of the incubation period. On the last day for all variants, a total methane yield between 3.66 and 3.88 mmol was found. Compared to all tested methanogens, the phenyl acids had the lowest impact on *M. wolfei's* methanogenesis, and no significant differences could be found between the variants (Figure S1).

Although hydrogenotrophic methanogenesis was described to be more robust against phenyl acids (Prem et al. 2021; Cabrol et al. 2015; Prem et al. 2023; Prem et al. 2023), in the present study, a negative influence of phenyl acids on hydrogenotrophic methanogens could be demonstrated. The methane production of the mesophilically incubated methanogens *M. vannielii* and *M. hungatei* showed a higher sensitivity to the phenyl acids compared to thermophilic methanogens *M. thermophilus, M. thermoautotrophicus*, and *M. wolfei* , whereby the effect of PAA, PPA, and PBA on the methane production of *M. wolfei* was nearly negligible. The higher sensitivity of mesophilic hydrogenotrophs concurs with the findings of Prem et al. (2020). They could demonstrate in mixed cultures that during the exposure to phenyl acids under thermophilic conditions, hydrogenotrophic methanogenesis was predominant, while under mesophilic conditions, acetoclastic methanogenesis was more important. In this study, PBA had the greatest inhibitory effect on the methanogenesis of pure cultures across all tested methanogens, followed by PPA and PAA. This effect has also been observed before in mixed cultures (Prem et al. 2023) and concurs with the findings of Sierra‐Alvarez and Lettinga (1991), which could demonstrate that the toxicity to acetoclastic methanogens increased with increasing hydrophobicity of the aromatic compound.

### 3.2 Consumption of Electron Donor and Electron Equivalent Recovery

**FIGURE 2:**
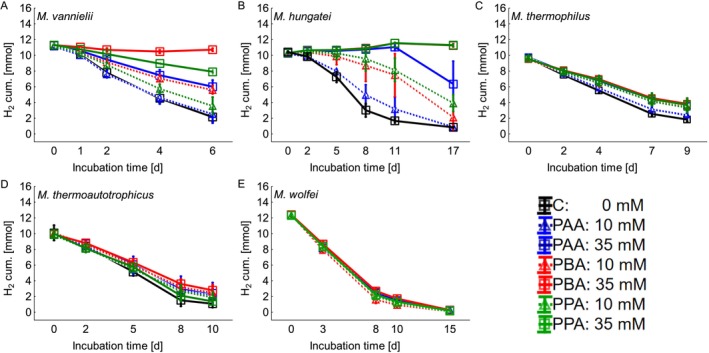
Cumulative consumption of the electron donator H_2_ during the exposure of phenyl‐acetic acid (PAA), phenyl‐butyric acid (PBA), and phenyl‐propionic acid (PPA) in different concentrations (0, 10, and 35 mM). A = *Methanococcus vannielii* , B = *Methanospirillum hungatei* , C = *Methanoculleus thermophilus*, D = *Methanothermobacter thermoautotrophicus*, and E = *Methanothermobacter wolfei*.

The cumulative consumption of the electron donator H_2_ is shown in Figure 2. In *M. vannielii* (Figure 2A) it was almost completely congruent with the cumulative methane production (Figure 1A). The 10 mM PAA variant showed the same kinetics in the consumption of H_2_ as the control, and in both variants, slightly more than 2 mmol H_2_ was left on the last incubation day. The 10 mM PPA variant showed a slight delay and still had 3.5 mmol H_2_ left on the last incubation day. Interestingly, already the methane production was slightly delayed in the 10 mM PPA variant, but it still reached the same methane yield as the control until the last incubation day (Figure 1A). By contrast, the consumption of H_2_ was also slightly delayed, but it still contained 1.5 mmol more H_2_ on the last incubation day compared to the control variant. The addition of 10 mM PBA and 35 mM PAA also resulted in a delay in H_2_ consumption in both variants, and approximately 6 mmol of H_2_ remained untouched until the last incubation day. In the 35 mM PPA variant, the consumption of H_2_ was strongly reduced, and still, 8 mmol of H_2_ were left at the end of the incubation period. 35 mM PBA inhibited the consumption of H_2_ the most, and more than 10 mmol of H_2_ could be evidenced on the last incubation day, which is consistent with the low methane formation rate in this variant (Figure 1A). The control of *M. vannielii* was able to recover 64.2% of the calculated electron equivalents (e–q) until incubation day 4 (Table 1). In the 10 mM PAA variant, slightly less e–q could be recovered at 62.6%, followed by 10 mM PPA at 54.4%, 10 mM PBA at 43.4%, 35 mM PAA at 39.9%, and 35 mM PPA at 28.1%. The lowest amount of e–q could be recovered in the 35 mM PBA variant at 7.4%.

**TABLE 1:** Electron equivalent recovery in the produced methane is shown as percentage of the initial substate (formate and H_2_‐CO_2_).

Organisms	Variant	Electron equivalent recovery (%)
*Methanococcus vannielii*	Control	64.2 ± 3.10
*Methanococcus vannielii*	PAA 10 mM	62.6 ± 4.25
*Methanococcus vannielii*	PAA 35 mM	39.9 ± 6.71
*Methanococcus vannielii*	PBA 10 mM	43.4 ± 2.26
*Methanococcus vannielii*	PBA 35 mM	7.4 ± 1.63
*Methanococcus vannielii*	PPA 10 mM	54.5 ± 4.45
*Methanococcus vannielii*	PPA 35 mM	28.1 ± 1.50
*Methanospirillum hungatei*	Control	72.0 ± 1.95
*Methanospirillum hungatei*	PAA 10 mM	66.0 ± 7.55
*Methanospirillum hungatei*	PAA 35 mM	2.0 ± 0.63
*Methanospirillum hungatei*	PBA 10 mM	27.8 ± 21.18
*Methanospirillum hungatei*	PBA 35 mM	0.6 ± 0.04
*Methanospirillum hungatei*	PPA 10 mM	19.4 ± 16.69
*Methanospirillum hungatei*	PPA 35 mM	0.3 ± 0.14
*Methanoculleus thermophilus*	Control	69.2 ± 2.61
*Methanoculleus thermophilus*	PAA 10 mM	70.0 ± 3.76
*Methanoculleus thermophilus*	PAA 35 mM	60.1 ± 2.36
*Methanoculleus thermophilus*	PBA 10 mM	58.8 ± 3.27
*Methanoculleus thermophilus*	PBA 35 mM	55.1 ± 5.24
*Methanoculleus thermophilus*	PPA 10 mM	60.0 ± 2.25
*Methanoculleus thermophilus*	PPA 35 mM	55.7 ± 5.09
*Methanothermobacter thermoautotrophicus*	Control	91.9 ± 3.39
*Methanothermobacter thermoautotrophicus*	PAA 10 mM	86.4 ± 6.51
*Methanothermobacter thermoautotrophicus*	PAA 35 mM	81.4 ± 6.76
*Methanothermobacter thermoautotrophicus*	PBA 10 mM	84.4 ± 5.13
*Methanothermobacter thermoautotrophicus*	PBA 35 mM	77.2 ± 5.43
*Methanothermobacter thermoautotrophicus*	PPA 10 mM	85.5 ± 5.23
*Methanothermobacter thermoautotrophicus*	PPA 35 mM	82.9 ± 4.65
*Methanothermobacter wolfei*	Control	94.3 ± 6.48
*Methanothermobacter wolfei*	PAA 10 mM	97.9 ± 6.50
*Methanothermobacter wolfei*	PAA 35 mM	94.3 ± 5.59
*Methanothermobacter wolfei*	PBA 10 mM	99.7 ± 4.27
*Methanothermobacter wolfei*	PBA 35 mM	93.3 ± 5.42
*Methanothermobacter wolfei*	PPA 10 mM	94.3 ± 7.32
*Methanothermobacter wolfei*	PPA 35 mM	95.6 ± 3.58

In *M. hungatei* , the cumulative consumption of H_2_ (Figure 2B) was more or less consistent with the methane production (Figure 1B). 10 mM PAA had the least impact on H_2_ consumption and only led to a slight delay compared to the control. Both 10 mM PAA and the control contained less than 1 mmol H_2_ on the last incubation day. The utilisation of H_2_ in the variants with 10 mM PPA and PBA was also delayed, and approximately 2 and 4 mmol H_2_ could be evidenced at the end of the experiment. With the addition of 35 mM PAA, the consumption of H_2_ was completely inhibited at the beginning and started, alike methanogenesis, on day 8 (Figure 1B). On the last incubation day, still more than 6 mmol of H_2_ were left. In the 35 mM PPA and PBA variant, the utilisation of H_2_ was completely inhibited throughout the whole incubation period, ruling out methanogenesis (Figure 1B). This indicates that 35 mM of PPA and PBA prevent *M. hungatei* from metabolic activity. Without phenyl acid addition, *M. hungatei* was able to recover 72% of the e–q until incubation day 8 (Table 1). When 10 mM PAA was added, 66% of the e–q could be recovered. In the 10 mM PBA and PPA variants, 27.8% and 19.4% could still be recovered, whereas in all the 35 mM variants hardly any e–q were recovered in methane until day 8. With 35 mM PAA, 2% of the e–q were recovered, and with 35 mM PBA and PPA only 0.6% and 0.3% were recovered, respectively. As was already evident in methane production (Figure 1B) and H_2_ consumption (Figure 2B) 35 mM of the tested phenyl acids are devastating for the metabolism of *M. hungatei* .

The impact of the phenyl acids on the methanogenesis of the thermophilic *M. thermophilus* was rather low (Figure 1C), and the same could be observed with the utilisation of H_2_ (Figure 2C). 10 mM PAA had the least effect on *M. thermophilus* , and this variant was most similar to the control. Starting at day 2, a slight delay in the consumption of H_2_ could be observed, and 2.3 mmol of H_2_ could be detected on the last incubation day, compared to 1.8 mmol of the control. In the other variants, H_2_ utilisation was inhibited slightly more compared to the 10 mM PAA variant, and a delay was observed, which increased slightly during the incubation period. The H_2_ content in these variants was between 3.5 and 4 mmol at the end of the incubation period. With *M. thermophilus* , the e–q recovery in the 10 mM PAA variant (70%) was slightly higher than in the control (69.2%) (Table 1). When 35 mM PAA was added, 60.1% of the e–q were recovered, followed by the 10 mM PPA variant at 60%, 10 mM PBA at 58.8%, 35 mM PPA at 55.7%, and 35 mM PBA at 55.1%. The impact of the added phenyl acids on the metabolism of *M. thermoautotrophicus* was also small, and the utilisation of H_2_ (Figure 2D) in most parts was congruent with the cumulative methane production (Figure 1D). Interestingly, 35 mM PPA had the least effect on the consumption of H_2_ of *M. thermoautotrophicus*, and on the last incubation day only slightly more H_2_ (1.4 mmol) could be evidenced than in the control (1.1 mmol). However, the negative effect of the other phenyl acid variants was only slightly higher, and the H_2_ content in these variants was between 1.9 and 2.8 mmol at the end of the incubation period. In *M. thermoautotrophicus*, 91.9% of the e–q could be recovered when no phenyl acids were added (Table 1). With 10 mM PAA, 86.4% of the e–q were recovered in methane, followed by 10 mM PPA at 85.5%, 10 mM PBA at 84.4%, 35 mM PPA at 82.9%, 35 mM PAA at 81.4%, and 35 mM PBA at 77.2%. *M. wolfei* showed high resistance to the phenyl acids, and no effect of the phenyl acids on the consumption of H_2_ (Figure 2E), as well as on methanogenesis (Figure 1E), could be proven. Until day 8, all variants depleted the major part of added H_2_ and less than 0.5 mmol remained at the end of the experiment. The most e–q were recovered when 10 mM PBA was added at 99.7%, followed by 10 mM PAA at 97.9%. All the other variants including the control recovered between 95.6% and 93.3% of the e–q (Table 1).

### 3.3 Assessment of Growth

**FIGURE 3:**
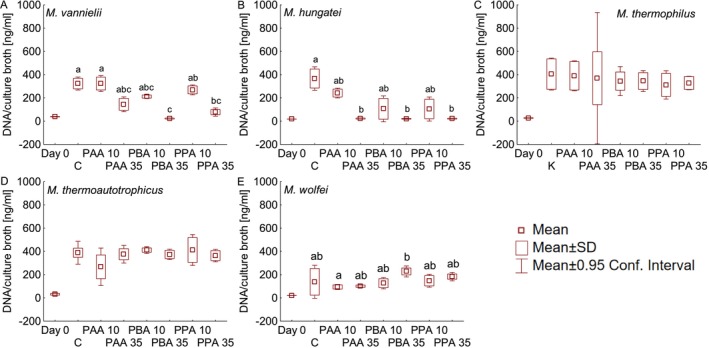
DNA content of the day when the respective control variant reached the maximum methane yield and the start of the incubation period (Day 0). Significant differences (p < 0.05) are indicated by various characters. A = *Methanococcus vannielii* (Kruskall–Wallis ANOVA and multiple comparison), B = *Methanospirillum hungatei* (Kruskall–Wallis ANOVA and multiple comparison), C = *Methanoculleus thermophilus* (one‐way ANOVA), D = *Methanothermobacter thermoautotrophicus* (one‐way ANOVA), and E = *Methanothermobacter wolfei* (Kruskall–Wallis ANOVA and multiple comparison).

DNA concentration was shown to be a suitable parameter to assess microbial growth in anaerobic systems (Malin and Illmer 2008) and was therefore used in this study to quantify the impact of phenyl acids on microbial growth. DNA from day 0 and from the day when the respective control variant reached maximum methane concentration was extracted and is depicted in Figure 3. Regarding *M. vannielii* (Figure 3A), all variants started with a DNA concentration of 33.99 ± 5.252 ng/mL. In the control and the 10 mM PAA variant, approximately 320 ng DNA/mL culture broth could be determined, being significantly higher than in the 35 mM PBA (23 ng/mL) and the 35 mM PPA variant (78 ng/mL). In the 10 mM PPA variant, 269 ng/mL DNA could be proven to be significantly more than in that with 35 mM PBA. However, no significant differences could be determined between 10 mM PBA (211 ng/mL), 35 mM PAA (145 ng/mL), and all other variants.

In *M. hungatei* (Figure 3B) the DNA concentration on day 0 was on average 16.51 ± 3.097 ng/mL. In controls, 366 ng DNA/mL could be confirmed, significantly more than in all 35 mM variants (PAA, PBA, and PPA), which were all between 20 and 23 ng DNA/mL. Interestingly, although considerably more methane was produced in the 35 mM PAA variant than in the 35 mM PBA and PPA variants (Figure 1B), the DNA content was rather similar. 10 mM PAA had the least effect on the growth of *M. hungatei* , with 242 ng/mL being detected, whereas in the 10 mM PBA and PPA variants, approximately 105 ng/mL could be evidenced, not being significantly different from all other variants.

In *M. thermophilus* , all variants contained 32.73 ± 7.224 ng/mL on day 0 (Figure 3C). The control variant showed a DNA concentration of 405 ng DNA/mL, followed by 10 mM PAA with 390 ng/mL, 35 mM PAA with 370 ng/mL, 10 and 35 mM PBA with 345 ng/mL, 35 mM PPA with 328 ng/mL, and 10 mM PPA with 312 ng/mL. No significant differences could be found between all variants, and the obtained concentrations were the highest among the tested methanogens.

The DNA concentration in *M. thermoautotrophicus* on day 0 was on average 33.34 ± 7.737 (Figure 3D), while the highest DNA concentration was not found in the control but in the 10 mM PPA variant with 412 ng/mL and 10 mM PBA with 411 ng/mL. The control showed a DNA concentration of 388 ng/mL, whereas for all other variants, values between 376 and 364 ng DNA/mL were found, except for 10 mM PAA with 267 ng/mL. However, significant differences reflecting an impact of phenyl acids on growth with regard to cells' DNA concentration could not be secured.

The average DNA concentration in *M. wolfei* cultures on day 0 was 20.63 ± 0.632 ng/mL (Figure 3E). Interestingly, for *M. wolfei* , the 35 mM PBA variant had the highest DNA concentration of all variants, with 228 ng/mL being significantly higher than that of 10 mM PAA (94 ng/mL). However, no significant differences could be found between all other variants, achieving between 183 ng ml (35 mM PPA) and 94 ng/mL (10 mM PAA).

### 3.4 Turnover of Volatile Fatty Acids

**FIGURE 4:**
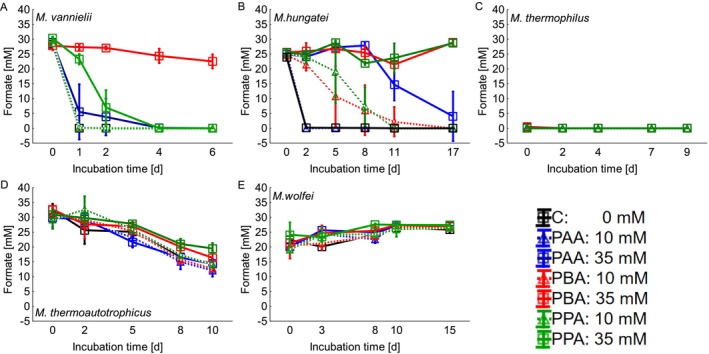
Formate concentration during the exposure of phenyl acetic acid (PAA), phenyl butyric acid (PBA), and phenyl propionic acid (PPA) in different concentrations (0, 10, and 35 mM). A = *Methanococcus vannielii* , B = *Methanospirillum hungatei* , C = *Methanoculleus thermophilus* , D = *Methanothermobacter thermoautotrophicus, and E = Methanothermobacter wolfei*.

*M. vannielii* possesses a selenium‐dependent formate dehydrogenase (Jones et al. 1979; KEGG: Kyoto Encyclopedia of Genes and Genomes n.d.‐a), it can utilise formate for methanogenesis next to H_2_‐CO_2_. The control and all 10 mM variants of *M. vannielii* depleted formate completely within the first day, indicating the high affinity of *M. vannielii* towards formate (Figure 4A). In contrast, the 35 mM PAA and PPA variants consumed it until day 4, and the 35 mM PBA variant had still more than 20 mM formate left on the last incubation day. The low turnover rate in the 35 mM PBA variant is consistent with the low methane formation rate (Figure 1A). Interestingly, the methane production in the 35 mM PAA variant was very similar to that of 10 mM PBA (Figure 1A), whereas the turnover rate of formate was much faster in the 10 mM PBA variant (Figure 4A).

Via a formate dehydrogenase (KEGG: Kyoto Encyclopedia of Genes and Genomes n.d.‐b) *M. hungatei* is able to use formate for methanogenesis. Control flasks of *M. hungatei* and those with 10 mM PAA completely utilised formate within 2 days (Figure 4B). The 10 mM PPA and PBA variants showed a strong delay in the consumption of formate but were able to consume it until day 11 (10 mM PPA) and 17 (10 mM PBA), respectively. The 35 mM PPA variant started with the utilisation of formate on day 8, thus at the same time when methane formation began (Figure 1B). In contrast, the 35 mM PBA and PPA variants could not use formate until the end of the incubation period, and methane formation in these variants never started (Figure 1B).

*M. thermophilus* CR‐1 (DSM 2373) was shown to grow on formate (Rivard and Smith 1982), but the Methanogenium medium 141, which is the suggested cultivation medium by DSMZ (in this study for all organisms the suggested media were used to secure optimum growth conditions), does not contain formate, and only traces could be evidenced in some variants (Figure 4C).

According to Wasserfallen et al. (2000), *Methanothemobacter thermoautotrophicus* ∆H (DSM 1053, used in this study) cannot grow on formate, while other strains, for example, Z‐245 (DSM 3720, not used in this study) can. However, according to the KEGG database, *Methanothemobacter thermoautotrophicus* ∆H (DSM 1053) possesses a formate dehydrogenase and also a formate hydrogenlyase (KEGG: Kyoto Encyclopedia of Genes and Genomes n.d.‐c) and should be able to convert formate. In our study, a decrease in formate concentration could be observed in all variants and on the last incubation day, slightly less than 15 mM formate could be evidenced, except for the 35 mM PBA and PPA variants, which had between 15 and 20 mM (Figure 4D). *M. wolfei* (DSM 2970) can grow on formate (Wasserfallen et al. 2000). However, a decrease in formate could not be observed in any of the variants in the present investigation (Figure 4E).

**FIGURE 5:**
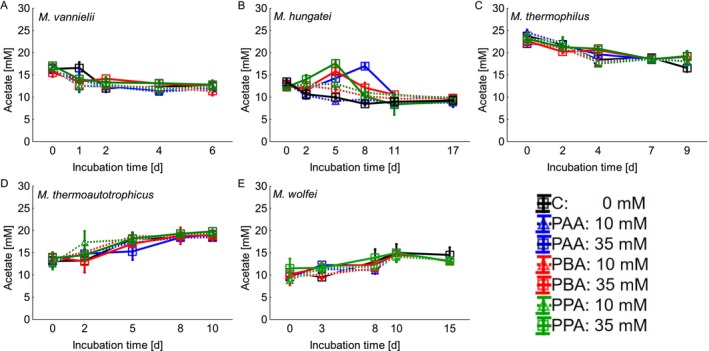
Acetate concentration during the exposure of phenyl‐acetic acid (PAA), phenyl‐butyric acid (PBA), and phenyl‐propionic acid (PPA) in different concentrations (0, 10, and 35 mM). A = *Methanococcus vannielii* , B = *Methanospirillum hungatei* , C = *Methanoculleus thermophilus* , D = *Methanothermobacter thermoautotrophicus*, and E = *Methanothermobacter wolfei*.

*M. vannielii* can grow on formate as the sole carbon source (Jones and Stadtman 1977) and does not require acetate for growth. However, all variants, except for the control, showed a decrease in acetate concentration from around 16 to 13 mM until incubation day 1 (Figure 5A). In the control, the acetate content remained stable until incubation day 1 and decreased to 12 mM on day 2. After that, no further decrease in acetate concentration was observed in any of the variants, and on the last incubation day, all variants had approximately 12 mM acetate.

*M. hunagtei* strain GP‐1 and JF‐1 require acetate for growth (Sprott and Jarrell 1981). In all variants, a degradation of acetate could be observed, and on the last incubation day, approximately 9 mM acetate could be evidenced in all variants (Figure 5B). In the control and 10 mM PAA variants, the acetate concentration decreased until incubation day 8 to subsequently remain stable, which is consistent with the methane production in these two variants (Figure 1B). The 10 mM PBA and PPA variants consumed the acetate slowly throughout the incubation period, a trend that was consistent with the methane production in these variants (Figure 1B). In the 35 mM PAA variant, the acetate concentration increased to 17 mM until day 8 and subsequently decreased when methane formation started in this variant (Figure 1B). Whereas in the 35 mM PBA and PPA variants, where methanogenesis was completely inhibited (Figure 1B), an increase in the acetate concentration could be observed until day 5, which, however, decreased in the following days.

Most strains of *M. thermophilus* require acetate for growth, but there are single strains that might be autotrophic (Spring et al. 2005). The consumption of acetate could be observed in all variants (Figure 5C), indicating the ability of the used strain, *M. thermophilus* CR‐1, to use acetate for assimilatory metabolism. The consumption of acetate in all variants was similar and decreased throughout the whole incubation period only slowly, and on the last incubation day, the acetate concentration was found to be between 16 and 19 mM.

*M. thermoautotrophicus* ssp. was shown to grow autotrophically and not to require acetate for growth (Zeikus and Wolfe 1972). In all variants, an increase of acetate could be observed (Figure 5D) from about 13 mM at the beginning of the incubation period to approximately 19 mM at the end. *M. wolfei* is capable of fixing CO_2_ for cell carbon and therefore able to grow autotrophically (Blotevogel et al. 1985). Similar to *M. thermoautotrophicus*, an increase in acetate concentrations could be observed in all variants (Figure 5E) from approximately 10 to 13 mM in the phenyl acid‐containing variants and 15 mM in the control variant.

## 4 Conclusion

Even though it was assumed in previous investigations that hydrogenotrophic methanogenesis in complex microbial communities was more robust against phenyl acids compared to acetoclastic methanogenesis, a negative influence of phenyl acids on hydrogenotrophic methanogens in pure culture could be proven in this study. Increased sensitivity against phenyl acids was observed for mesophilic methanogens, therefore suggesting that temperature plays a crucial role regarding the effect of the phenyl acids on the investigated Archaea. Regarding the investigated methanogens *M. vannielii* SB (DSM 1224), *M. hungatei* JF‐1 (DSM 864), *M. thermophilus* (DSM 2373), *M. thermoautotrophicus* Delta H (DSM 1053), and *M. wolfei* (2970), PBA had the greatest adverse impact on methanogenesis, followed by PPA and PAA. However, thermophilic organisms' ability to produce methane was merely impacted by the presence of up to 35 mM phenyl acids. In further investigations, it has to be clarified if and how other microorganisms involved in the AD process are influenced by the phenyl acids. Additionally, it has to be figured out how phenyl acids act on microorganisms and how they negatively affect their metabolism. A more detailed knowledge will help in the highly complex trophic interaction system of anaerobic digestion to better understand disturbances caused by degradation products, an urgent need if increased amounts of lignocellulose‐ and protein‐rich substrates are used for biogas production to ensure a stable AD process.

## Author Contributions

**Mathias Wunderer:** investigation, data curation, conceptualization, visualization, methodology, writing – original draft, writing – review and editing. **Martin Unterkircher:** investigation, data curation. **Anna Schmidhofer:** investigation, data curation. **Eva Maria Prem:** validation, writing – review and editing. **Andreas O. Wagner:** validation, resources, writing – review and editing, supervision, project administration, funding acquisition.

## Conflicts of Interest

The authors declare no conflicts of interest.

## Supporting information


**Figure S1.** Box plot of the cumulative methane production from the day when the control variant reached the maximum methane yield. Significant differences (*p* < 0.05) are indicated by various characters. A = *Methanococcus vannielii* : incubation day 4 with one‐way ANOVA and Bonferroni post hoc test. B = *Methanospirillum hungatei* : incubation day 8 with Kruskall–Wallis ANOVA and multiple comparison. C = *Methanoculleus thermophilus* : incubation day 7 with one‐way ANOVA and Bonferroni post hoc test. D = *Methanothermobacter thermoautotrophicus*: incubation day 8 with Kruskall–Wallis ANOVA. E = *Methanothermobacter wolfei*: incubation day 15 with one‐way ANOVA.

## Data Availability

The data that support the findings of this study are available on request from the corresponding author.
