# Supplementary material for: Phenyl Acid Induced Inhibition of Methanogenesis in CO2 Reducing Organisms
Source: Environ Microbiol Rep. 2025 Mar 12;17(2):e70082. doi: 10.1111/1758-2229.70082 (PMC11903324; doi:10.1111/1758-2229.70082)
Supplement: Supplementary file 1 — Figure S1. Box plot of the cumulative methane production from the day when the control variant reached the maximum methane yield. Significant differences (p < 0.05) are indicated by various characters. A = Methanococcus vannielii : incubation day 4 with one‐way ANOVA and Bonferroni post hoc test. B = Methanospirillum hungatei : incubation day 8 with Kruskall–Wallis ANOVA and multiple comparison. C = Methanoculleus thermophilus : incubation day 7 with one‐way ANOVA and Bonferroni post hoc test. D = Methanothermobacter thermoautotrophicus: incubation day 8 with Kruskall–Wallis ANOVA. E = Methanothermobacter wolfei: incubation day 15 with one‐way ANOVA. [file EMI4-17-e70082-s001.docx]

Figure S1: Box plot of the cumulative methane production from the day when the control variant reached the maximum methane yield. Significant differences (p < 0.05) are indicated by various characters. A = Methanococcus vannielii: incubation day 4 with one-way ANOVA and Bonferroni post-hoc test. B = Methanospirillum hungatei: incubation day 8 with Kruskall-Wallis ANOVA and multiple comparison. C = Methanoculleus thermophilus: incubation day 7 with one-way ANOVA and Bonferroni post-hoc test. D = Methanothermobacter thermoautotrophicus: incubation day 8 with Kruskall-Wallis ANOVA. E = Methanothermobacter wolfei: incubation day 15 with one-way ANOVA.
